# Circuit Optimization Method to Reduce Disturbances in Poly-Si 1T-DRAM

**DOI:** 10.3390/mi12101209

**Published:** 2021-10-02

**Authors:** Yejin Ha, Hyungsoon Shin, Wookyung Sun, Jisun Park

**Affiliations:** 1 Department of Electronic and Electrical Engineering, Ewha Womans University, Seoul 03760, Korea; jassie0627@ewhain.net (Y.H.); hsshin@ewha.ac.kr (H.S.); 2 Graduate Program in Smart Factory, Ewha Womans University, Seoul 03760, Korea; 3 Department of Electrical and Computer Engineering, Seoul National University, Seoul 08826, Korea

**Keywords:** capacitorless one-transistor dynamic random-access memory, 1T-DRAM, polysilicon, array, circuit, memory

## Abstract

A capacitorless one-transistor dynamic random-access memory device (1T-DRAM) is proposed to resolve the scaling problem in conventional one-transistor one-capacitor random-access memory (1T-1C-DRAM). Most studies on 1T-DRAM focus on device-level operation to replace 1T-1C-DRAM. To utilize 1T-DRAM as a memory device, we must understand its circuit-level operation, in addition to its device-level operation. Therefore, we studied the memory performance depending on device location in an array circuit and the circuit configuration by using the 1T-DRAM structure reported in the literature. The simulation results show various disturbances and their effects on memory performance. These disturbances occurred because the voltages applied to each device during circuit operation are different. We analyzed the voltage that should be applied to each voltage line in the circuit to minimize device disturbance and determine the optimized bias condition and circuit structure to achieve a large sensing margin and realize operation as a memory device. The results indicate that the memory performance improves when the circuit has a source line and the bias conditions of the devices differ depending on the write data at the selected device cell. Therefore, the sensing margin of the 1T-DRAM used herein can expectedly be improved by applying the proposed source line (SL) structure.

## 1. Introduction

With conventional one-transistor one-capacitor random-access memory (1T-1C-DRAM), there is a limit on the extent to which the device density can be increased because as the amount of data we use increases, the size of electronic devices decreases while the absolute physical space is limited. Therefore, capacitorless one-transistor dynamic random-access memory device (1T-DRAM), which does not need capacitor fabrication, has emerged as an alternative to conventional 1T-1C-DRAM [1,2,3,4,5]. In recent years, among the various types of 1T-DRAM, poly-Si 1T-DRAM is being researched actively [5,6,7,8,9,10,11]. Poly-Si 1T-DRAM exhibits small degradation over short channels because it can operate as memory in fully depleted silicon-on-insulator (FD-SOI) structures using grain boundary (GB) [10,11,12].

Studies on poly-Si 1T-DRAM devices have focused on overcoming the limitations of conventional silicon 1T-DRAM devices and improving memory performance, and therefore, the focus has been on device-level simulation [4,5,6,7,8,9,10,11,12,13]. Although it is important for a single 1T-DRAM device to have adequate performance as memory, a single device cannot be used as memory. Therefore, circuit-level studies are essential to facilitate the substitution of 1T-1C-DRAM with 1T-DRAM. The operating method of 1T-DRAM is different from that of conventional 1T-1C-DRAM, for example, the use of band-to-band tunneling (BTBT) as are the bias conditions [2,11,14]. Thus, the operation of 1T-DRAM cannot be verified using the conventional 1T-1C-DRAM circuit structure. It is important to confirm through circuit-level research whether problems exist, such as degradation of memory performance, and to identify an optimized 1T-DRAM circuit structure. Based on the results of the circuit-level research, we can control the disturbances that occur in 1T-DRAM more effectively.

In this paper, we investigate the optimized poly-Si 1T-DRAM structures proposed in the literature. In Section 2, we define the circuit structure and name used in this paper depending on the circuit location, in addition to defining disturbance. In Section 3, we present simulation results. First, we perform a simulation with the bias condition used in the previous device-level study. To solve the unexpected problems encountered in the device-level simulation, a different optimized circuit structure is needed. Therefore, we perform a second simulation with an improved circuit structure and bias condition. Additionally, the sensing margin is expressed in the form of a graph as a function of the size and structure of the array circuit. 

## 2. Structures and Methods

**Figure 1:**
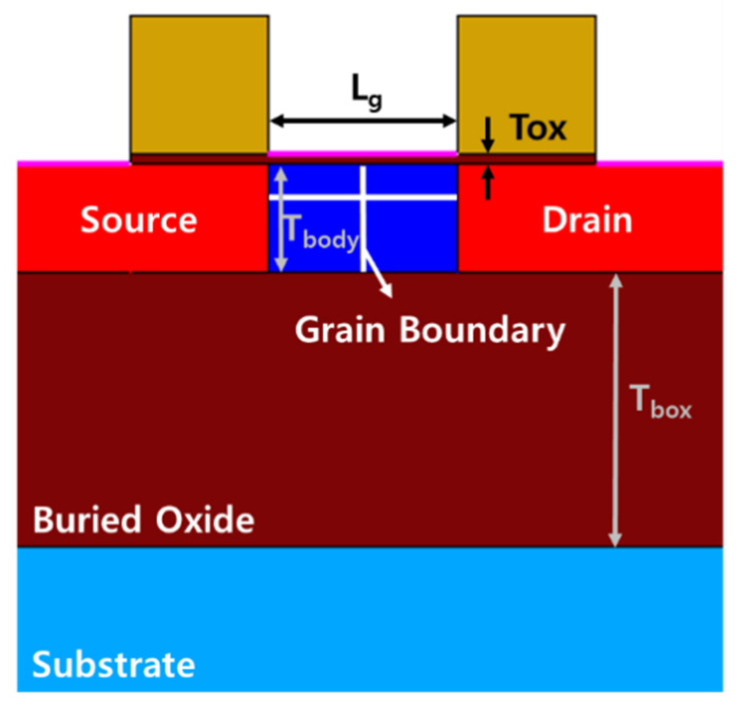
Simulated structure of poly-Si 1T-DRAM cell with a lateral GB and vertical GB.

**Figure 2:**
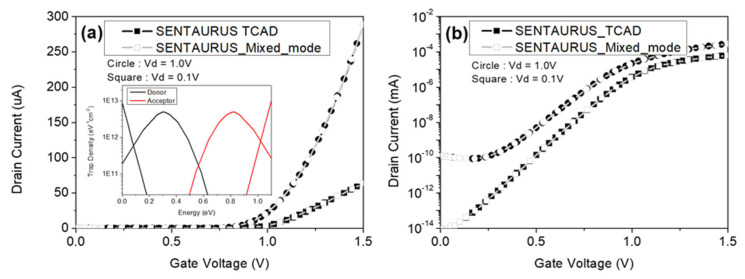
Transfer curve of poly-Si 1T-DRAM cell in simulations with (**a**) linear scale (inset, trap densities used in the simulations) and (**b**) log scale.

In this study, we performed simulations by using the mixed-mode simulator built into the SENTAURUS^TM^ application to investigate the changes within the circuit devices proposed in a previous study on 1T-DRAM. Figure 1 shows the structure of the poly-Si 1T-DRAM used in the simulation. The device structure is composed of one vertical grain boundary (GB) and one lateral GB, and it was optimized by conducting a device-level memory performance evaluation. The device parameters were the same as those used in the reference paper [15]. In the reference study, the memory performance of the device structure was evaluated using a SENTAURUS^TM^ TCAD simulator, while in the present study, it was evaluated using a SENTAURUS^TM^ mixed-mode simulator. Because different simulators were used in the two studies, before starting the simulation, we confirmed that the device had the same characteristics in the two simulators. Figure 2a,b shows that the device characteristics in the two simulators are the same on the log scale and the linear scale, respectively. The inset graph of Figure 2a shows the density of states distribution of the GB. The basic circuit structure used in the simulation was the 3 × 3 array structure, and the unit for storing one bit of data was called a cell. The cells themselves were named unselected cell, shared bit line (BL) cell, shared word line (WL) cell, and selected cell depending on their locations in the circuit. Additionally, the circuit line resistance was set to zero to analyze the within-device variations of each cell depending on the applied voltage shared with the selected cell. When data were written to one cell (selected cell), deformation of the data stored in the other cells, that is, change in read current (drain current), was termed a disturbance. Disturbances “0” and “1” indicate that the read currents of the other cells were changed by the applied voltage when “0” or “1” was written onto the selected cell, respectively. To analyze all cases of disturbance, we simulated four cases, as summarized in Table 1. The first write occurs in all cells, and the second write occurs only in the selected cell. 

## 3. Results and Discussion

This section shows the results and discussion of the two circuit structure. The first section analyzes structure without a source line (SL) case and the second section analyzes structure with an SL case. As a result of the analysis, the sensing margin is improved by approximately 60% in the second case compared to the first case.

### 3.1. Simulation without SL

**Figure 3:**
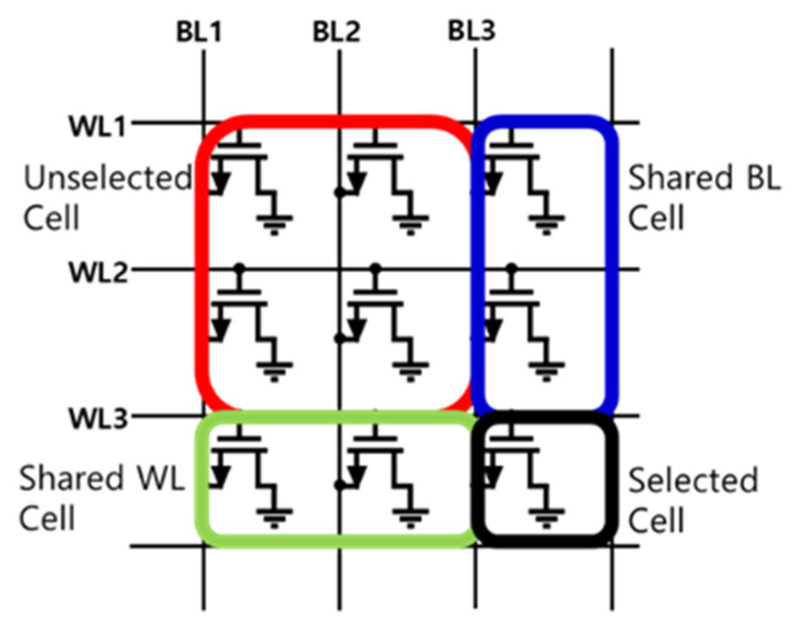
Array circuit structure and cell names used in first simulation.

**Figure 4:**
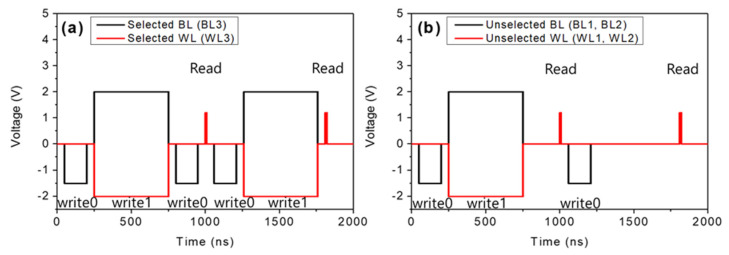
Timing diagram of first simulation for (**a**) selected WL and BL and (**b**) unselected WL and BL.

**Figure 5:**
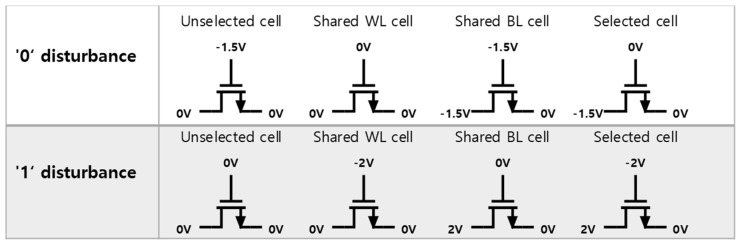
Voltage applied at each cell location depending on the type of disturbance in the first simulation.

Figure 3 shows the array structure used in the first simulation. It consists of a cell for storing data, a WL connecting each gate terminal of transistors to each other, and a BL connecting each drain terminal of transistors to each other so that the same line shares the applied voltage. The selected cell in this study was a (3, 3) cell. The bias conditions used and the corresponding pulse timing diagrams are summarized and shown in Table 2 and Figure 4, respectively. The sample timing diagram presented in Figure 4 is expressed only for the “1”-“0” case and “0”-“1”. For removing the remaining trapped holes at the GB, in all of the simulation cases, we initially wrote “0” on all cells before the first write. Figure 4a shows the pulses applied to the selected line, that is, the pulses applied to BL3 and WL3. Figure 4b shows the pulses applied to the unselected line, that is, to BL1, BL2, WL1, and WL2, except for the BL3 and WL3. Although not shown in Figure 4, the hold operation is performed once before and after each write and read operation. Figure 5 shows the voltages applied at each cell location depending on the data written to the selected cell. Because the voltage line shared with the selected cell is different for each cell, the applied voltage is different as well. For this reason, it can be expected that the disturbance will differ depending on the cell location.

**Figure 6:**
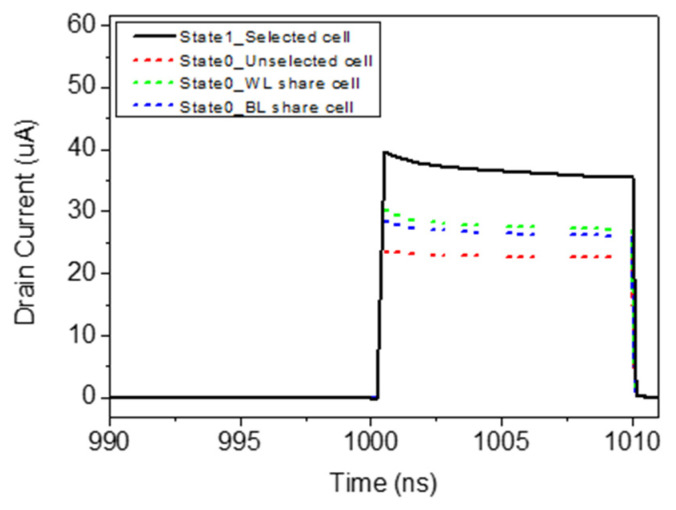
Read current for “0”-“1” case (example of disturbance).

Figure 6 shows an example of disturbance in simulation. The “0”-“1” case is illustrated, and the figure shows the read current depending on the cell location. The solid and dotted lines indicate the read current of the “1” and “0” states, respectively. As illustrated in Figure 5, the three dotted lines represent different current levels owing to different degrees of disturbance. As such, in each of the four cases, only the cell with the greatest disturbance is selected and compared for measuring the sensing margin of the circuit. This means that the “0” and “1” state cells with the highest “0” current and the lowest “1” current, respectively, are selected. The sensing margin is calculated as the difference between the lowest value of “1” current and the highest value of “0” current among the four cases. The current values used to calculate the sensing margin are the values at 10 ns after application of the read voltage.

**Figure 7:**
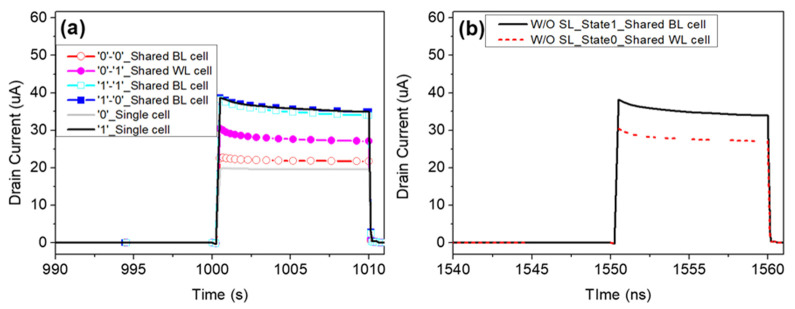
Read current of (**a**) worst cell for each case and single device and (**b**) worst cell for each state in all cases.

Figure 7a shows the read current of the cell with the largest disturbance in each of the cases. No symbol, circle symbol, and square symbol indicate the read currents of the data written to a single cell, largest disturbance among the “0” current cases, and largest disturbance among the “1” current cases, respectively. As depicted in Figure 5, in the “0”-“0” case, the “0” current disturbance of the shared BL cell is the largest owing to the BTBT on the drain side and the migration of trapped electrons due to the source–drain voltage difference (V_ds_). In the “0”-“1” case, the “0” current disturbance of the shared WL cell is the largest due to the BTBT on both the drain and source sides. In the “1”-“1” case, the “1” current disturbance of the shared BL cell is the largest because the trapped holes are removed upon the application of V_ds_. Finally, in the “1”-“0” case, there are no disturbances in any cell; therefore, the shared BL cell with the least improvement is selected. Figure 7b shows the read currents selected for calculating the sensing margin. Table 3 lists the drain current values measured at 10 ns after application of the read voltage and the calculated sensing margin values. Considering that the minimum sensing margin for distinguishing whether a data point is 0 or 1 in the circuit is 3 µA, the simulated array structure satisfies the minimum sensing margin condition, meaning that it can be used as a memory device [16].

### 3.2. Simulation with SL

**Figure 8:**
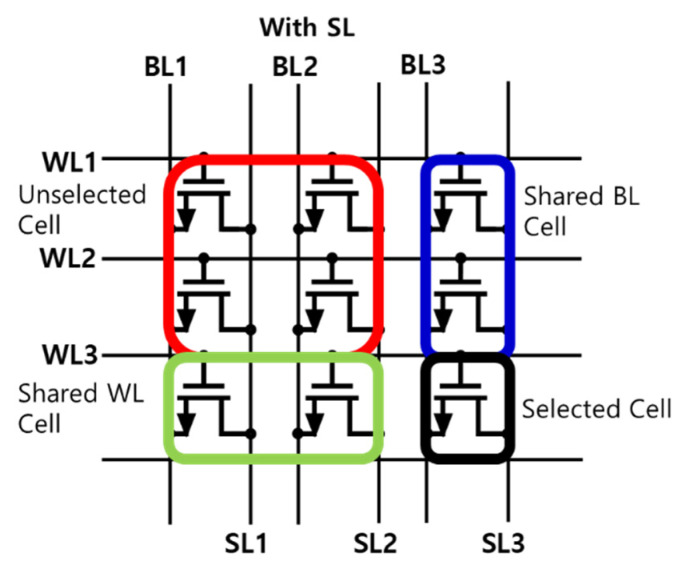
Array circuit structure and cell names used in second simulation.

**Figure 9:**
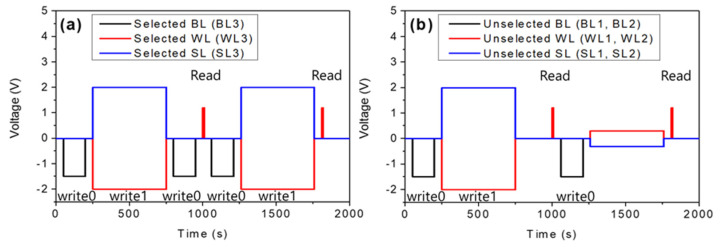
Timing diagram of second simulation for (**a**) the selected WL, BL, and SL and (**b**) unselected WL, BL, and SL.

**Figure 10:**
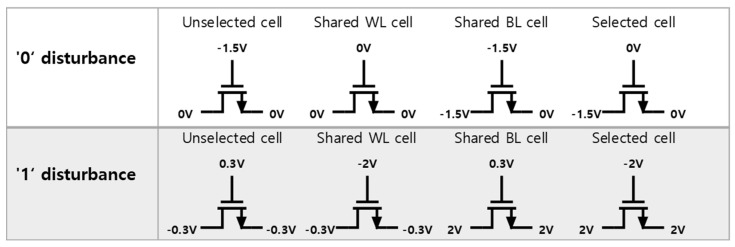
Voltage applied at each cell location depending on the type of disturbance in second simulation.

Although the sensing margin of 6.86 µA exceeds the minimum sensing margin threshold, stable operation of the previously proposed circuit is not possible with variables such as continuous disturbance or long hold time. Therefore, the sensing margin must be improved to realize stable circuit operation. To this end, increasing the “1” current is more effective than the decreasing the “0” current by controlling V_ds_. The “1” current is reinforced due to the occurrence of the BTBT on both the source and drain sides. To ensure the occurrence of the BTBT on the source side, 2 V is applied to source of the cell during the write “1” operation. Therefore, as shown in Figure 8, the source line (SL) connecting the source of the cell is formed in parallel with the BL. The line resistance and cell name depending on cell location are the same as those in the previous simulation. The colors of the boxes indicating the cell locations are the same as the colors representing the read currents of each of the locations in the Figure 9, Figure 12 and Table 4 show and summarize, respectively, the pulse and bias condition used in the simulation. The underlined text in Table 4 represents the part that is different relative to the bias condition used in the previous simulation. To achieve the maximum sensing margin, different voltages are applied to the unselected WL and BL during the write “1” and write “0” operations, as in the previous simulation, but because the SL is added, the bias condition of the unselected WL and BL during the write “1” operation (V_Unselected_1_) is optimized to ±0.3 V to arrive at the optimal bias condition. Moreover, 2 V is applied at the source, as at the drain, to realize the BTBT. Figure 9a,b show the pulses of the selected line (BL3, WL3, and SL3) and unselected line (BL1, BL2, WL1, WL2, SL1, and SL2), respectively. Moreover, as in Figure 4, Figure 9 shows only the pulses of the “1”-“0” and “0”-“1” cases as examples. Figure 10 shows the voltage applied at each cell location. The applied voltage is different compared to that in the first simulation due to the SL.

**Figure 11:**
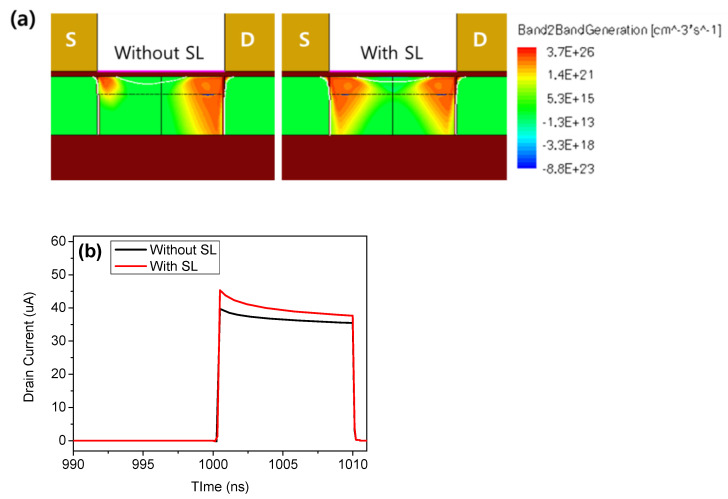
(**a**) BTBT generation contour of the proposed device with and without SL. (**b**) Difference in “1” current after one write “1” operation with and without SL.

Figure 11 shows the difference between the with- and without-SL cases about write “1” operation. Figure 11a shows the two-dimensional (2D) contour of the proposed 1T-DRAM. Considering that BTBT occurs on the source sides in the with-SL case but not on the source sides in the without-SL case, we can confirm that more holes are created in the with-SL case. Figure 11b shows that the “1” current in the with-SL case is approximately 2.5 µA higher than that in the without-SL case after one write “1” operation. It can be expected that the difference in the “1” current between the 1T-DRAM with and without SL will exceed 2.5 µA after multiple repetitions of the write “1” operation. Additionally, considering that the sensing margin in the previous simulation was 6.9 µA, an increase of 2.5 µA in the “1” current is significant. Therefore, the SL is required in the array structure to improve the memory performance of the device.

**Figure 12:**
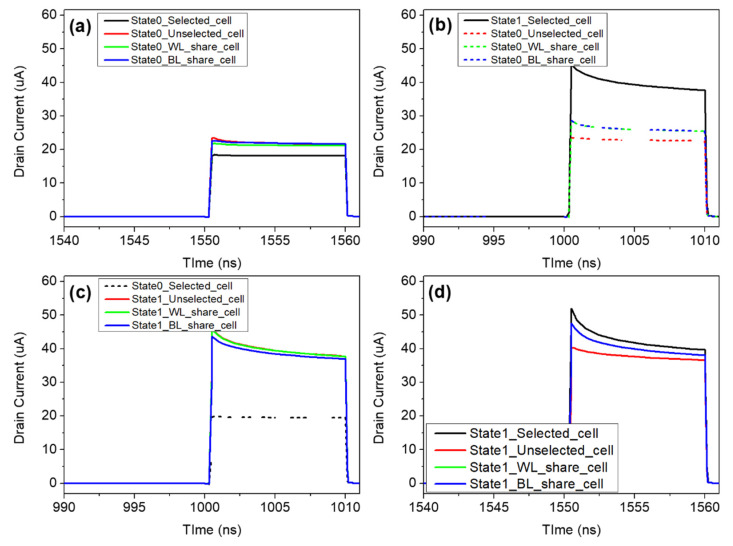
Read current of each cell location in (**a**) “0”-“0” case, (**b**) “0”-“1” case, (**c**) “1”-“0” case, and (**d**) “1”-“1” case.

Figure 12 and Table 5 show the read currents of the cells in each of the cases. Figure 12a shows the “0”-“0” case. The unselected cell and the shared BL cell have the largest disturbance, and the “0” current of the shared WL cell, where the applied voltage to all transistor terminals is 0 V, increases due to the retention of “0” current. Figure 12b shows the “0”-“1” case. The shared WL cell and the shared BL cell have the largest disturbance due to the occurrence of the BTBT, and as the disturbance time increases from 150 ns to 500 ns, the “0” current increases by approximately 4 µA compared to that in the “0”-“0” case. Therefore, the largest “0” current among all of the cases is that of the shared BL cell in the “0”-“1” case. Figure 12c shows the “1”-“0” case. Overall, the “1” currents of the cells increase compared to those in the previous simulation without the SL, and there is little “0” disturbance at any cell location. Figure 12d shows the “1”-“1” case. The “1” currents of the shared WL cell and the shared BL cell are higher due to the occurrence of the BTBT, and the unselected cell without enhancement has the least “1” current. Therefore, the least “1” current among all of the cases is that of the unselected cell in the “1”-“1” case.

**Figure 13:**
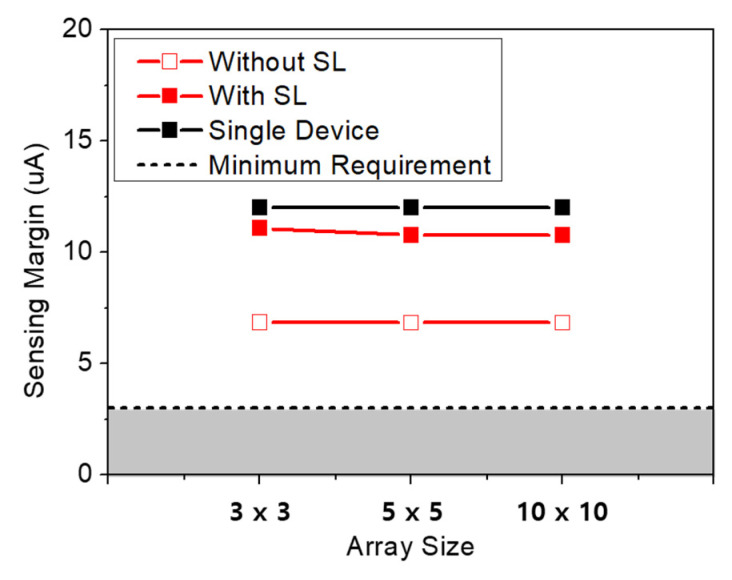
Sensing margin in the with- and without-SL cases according to array size, and minimum sensing margin for data identification.

Figure 13 and Table 6 show and summarize a comparison of the sensing margins in the with- and without-SL cases depending on the array size. The red solid lines represent the simulation data, the black solid lines represent single device performance of previous report, and the dashed line with gray boxes denotes the minimum required sensing margin [15]. As summarized in Table 6, the sensing margins are 6.86 µA, 6.84 µA, and 6.83 µA when the source is grounded without the application of any other voltage. The “0” current decreases by a lower margin than the “1” current. Additionally, the “1” current decreases continuously as the array size increases, and therefore, the sensing margin can be expected to decrease as the array size increases in the without-SL case. Meanwhile, after the addition of the SL to apply a voltage to the source, the sensing margins are 11.09 µA, 10.76 µA, and 10.76 µA. Unlike the without-SL case, there is almost no drop in the “1” current even with an increasing form 5 × 5 array to 10 × 10 array size and the reduction in sensing margin is only approximately 0.3 µA. This represents an increase of approximately 60% in all size of arrays. Additionally, in the with-SL case, the “1” current increases and the “0” current decreases compared to the without SL case, which is advantageous for data identification and disturbance control within the cell.

## 4. Conclusions

In this paper, we analyzed the memory performance of poly-Si 1T-DRAM and optimized the device array structure. The simulation was extended to circuit-level simulation, and the optimized device structure obtained in a previous device-level study was analyzed. The sensing margin of the initial simulation satisfied the required minimum sensing margin. However, the sensing margin decreased by approximately 50% compared to that in the device-level research [15]. Therefore, we proposed a new structure with an SL and evaluated the sensing margin characteristics of the proposed 1T-DRAM with the SL structure. When using the structure with an SL, the BTBT occurred on both the source and drain sides, more holes were created in the 1T-DRAM body, and the “1” current increased. Therefore, when disturbances occurred, the increase in the “1” current compensated for them and even improved the sensing margin. Thus, the sensing margin improved by 60% in all cases of the array compared to that of the without-SL structure. By using the verified with-SL structure, the stable data storage operation is expected even with large-sized arrays because of the improved sensing margin. Therefore, it is expected that the proposed 1T-DRAM can be used as a memory device. To analyze scenarios similar to those encountered in actual circuit operation, additional simulations involving tasks, such as inserting line resistance and increasing the array size, are required. Nevertheless, the results of this study will serve as an important guideline for circuit research on the use of 1T-DRAM as a memory device.

## Figures and Tables

**Table 1:** Simulated cases for each scenario.

1st-2nd Write	“0”-“0”	“0”-“1”	“1”-“1”	“1”-“0”
Unselected cell	State “0”	State “0”	State “1”	State “1”
Shared WL cell	State “0”	State “0”	State “1”	State “1”
Shared BL cell	State “0”	State “0”	State “1”	State “1”
Selected cell	State “0”	State “1”	State “1”	State “0”

**Table 2:** Bias and time conditions for transient operation of first simulation.

Operation	Write “1”	Write “0”	V_Unselected_1_	V_Unselected_0_	Read	Hold
V_g_ [V]	−2	0	0	−1.5	1.2	0
V_d_ [V]	2	−1.5	0	0	0.1	0
Time [ns]	500	150	500	150	10	50

**Table 3:** Read current values of the worst case in each state at 10 ns after application of the read voltage.

Worst Case in Each State	Drain Current [µA]
“1”-“1” case for shared BL cell	33.95
“0”-“1” case for shared WL cell	27.09
Sensing margin [µA]	6.86

**Table 4:** Bias and time conditions for transient operation in second simulation.

Operation	Write “1”	Write “0”	V_Unselected_1_	V_Unselected_0_	Read	Hold
V_g_ [V]	−2	0	0.3	−1.5	1.2	0
V_d_ [V]	2	−1.5	−0.3	0	0.1	0
V_s_ [V]	2	0	−0.3	0	0	0
Time [ns]	500	150	500	150	10	50

**Table 5:** Read current value at each location at 10 ns after application of the read voltage in the four cases.

Cell Name	Drain Current [µA]			
	“0”-“0” Case	“0”-“1” Case	“1”-“0” Case	“1”-“1” Case
Unselected cell	21.63	22.60	37.75	36.62
Shared WL cell	21.12	25.45	37.69	38.09
Shared BL cell	21.71	25.53	36.90	38.09
Selected cell	18.07	37.64	19.54	39.66

**Table 6:** Current values of states and sensing margin in the with- and without-SL cases according to array size.

Structure	Without SL			With SL		
Array Size	3 × 3	5 × 5	10 × 10	3 × 3	5 × 5	10 × 10
State “1” [µA]	33.95	33.90	33.89	36.62	36.29	36.29
State “0” [µA]	27.09	27.06	27.06	25.53	25.53	25.53
Sensing Margin [µA]	6.86	6.84	6.83	11.09	10.76	10.76
